# Repeated CT scans on 12,984 Asians to diagnose lung cancer once it is suspected

**DOI:** 10.3389/fonc.2024.1394402

**Published:** 2024-09-16

**Authors:** Vu Pham Thao Vy, Li-Nien Chien, Wan-Ting Chen, Wan-Ying Lin, Yeun-Chung Chang, Hsian-He Hsu, Wing P. Chan

**Affiliations:** ^1^ International PhD Program of Medicine, Taipei Medical University, Taipei, Taiwan; ^2^ Institute of Health and Welfare Policy, National Yang Ming Chiao Tung University, Taipei, Taiwan; ^3^ Health Data Analytics and Statistics Center, Office of Data Science, Taipei Medical University, New Taipei City, Taiwan; ^4^ Taiwan Radiological Society, Taipei, Taiwan; ^5^ Department of Radiology, National Taiwan University College of Medicine, Taipei, Taiwan; ^6^ Department of Radiology, Tri-Service General Hospital and National Defense Medical Center, Taipei, Taiwan; ^7^ Department of Radiology, School of Medicine, College of Medicine, Taipei Medical University, Taipei, Taiwan; ^8^ Department of Radiology, Wan Fang Hospital, Taipei Medical University, Taipei, Taiwan

**Keywords:** Asian, cancer screening, computed tomography (CT) scan, cost, lung cancer

## Abstract

In Taiwan, lung cancer remains the leading cause of cancer-related fatalities, resulting in substantial healthcare expenses. This research aims to evaluate both the frequency and the costs of low-dose computed tomography (LDCT) in individuals suspected of having lung cancer until their diagnosis of cancer. LDCT screening was not conducted on a population-wide scale, and asymptomatic participants had to cover the expenses for the screening personally or reimburse from other sources. If the screening results were positive or suspicious, National Health Insurance (NHI) could be utilized for subsequent follow-up examinations. This cohort study utilized the NHI Database and focused on individuals with suspected cases of lung cancer identified between 2010 and 2014. A total of 17,572 suspected new lung cancer cases were initially identified and assigned to the relevant International Classification of Diseases codes. Individuals with suspected lung cancer received a diagnosis following an average follow-up period of 2.24 (95%CI, 2.11-2.37) years, and required the use of 2.36 (95%CI, 2.20-2.51) repeated CT scans. The NHI expenditures incurred by the use of CT scans for monitoring suspected lung cancer cases were relatively modest.

## 1 Introduction

In Taiwan, lung cancer continues to be the primary cause of cancer-related deaths: in 2020, it resulted in 9629 deaths and 19.2% of all cancer-related deaths (1). The high incidence of lung cancer led to significant healthcare expenditures in Taiwan: in 2020, lung cancer care costs were USD $657.5 million or a per capita cost of around $7900 (2). Low-dose computed tomography (LDCT) has been shown to be an effective screening tool for lung cancer and to reduce mortality among those at high risk, particularly smokers (3, 4).

The US Preventive Services Task Force (USPSTF) recommends annual screening for lung cancer with LDCT in adults aged 55 to 80 years who have a 30–pack-year smoking history and currently smoke or have quit within the past 15 years (5). Age and smoking status are the primary drivers of lung cancer risk, with smoking accounting for nearly 90% of all lung cancer cases. Additional factors that contribute to the risk of lung cancer include environmental exposures, radiation therapy, other lung diseases not related to cancer, race/ethnicity, and family history.

However, the evidence in East Asia differs from that in other regions in that there are significantly more lung cancer patients who have never smoked (6, 7). Research has uncovered other risk factors that may be at play such as a family history of lung cancer, chemical exposure, poor cooking ventilation, and chronic lung diseases.

In Taiwan, lung cancer screening with LDCT is currently not covered by National Health Insurance (NHI). The NHI is a single-payer, compulsory fee-for-service health insurance program with a budget that extends coverage to about 99.6% of the population (8). Self-paying asymptomatic lung cancer screening is available, or since 2023, the Ministry of Health and Welfare has conducted biannual LDCT screening for smokers and families with lung cancer. The NHI can only be used for follow-up examinations if the screening results are positive or not conclusive. The aim of this study was to determine the frequency and cost of CT scans in patients with suspected lung cancer until their diagnosis.

## 2 Methods

This study was approved by the X Medical University-Joint Institutional Review Board (N202402019). Informed consent was waived due to the retrospective nature of the study. This retrospective cohort study was conducted using the Taiwan National Health Insurance (NHI) Database, enrolling those with new cases of suspected lung cancer from 2010 to 2014. During this period, the screening of LDCT was not a population-based program, and people should self-pay for the screening if they are asymptomatic. If screening results were positive or inconclusive, NHI covered follow up LDCT examinations. Data was collected with encryption of individual information and disease identification using the International Classification of Diseases code, ninth revision, clinical modification (ICD-9-CM). At the end of 2014, the NHI offered universal health insurance with broad coverage that included 99.6% of the Taiwanese population (8). From January 2010 to December 2014, the ICD-9-CM was used not only to identify suspected new lung cancer cases, but also to track the frequency of repeated CT scanning, with follow-up continuing until as late as 2018. We excluded individuals who were diagnosed with other cancers in the two years prior to study initiation (2008 and 2009) and lung cancers within 6 months. Lung cancer diagnoses were confirmed by the National Cancer Registry. After statistical analysis (SPSS version 18.0), data were tabulated and expressed as mean (standard deviation) in the cases of continuous variables and *n* in the cases of categorical variables. Comparisons were conducted using the independent *t* test for continuous variables and the chi-square test for categorical variables. Point estimates and their 95% confidence intervals (CIs) were also reported. Statistical significance was recognized when *p* < 0.05.

## 3 Results

**Figure 1:**
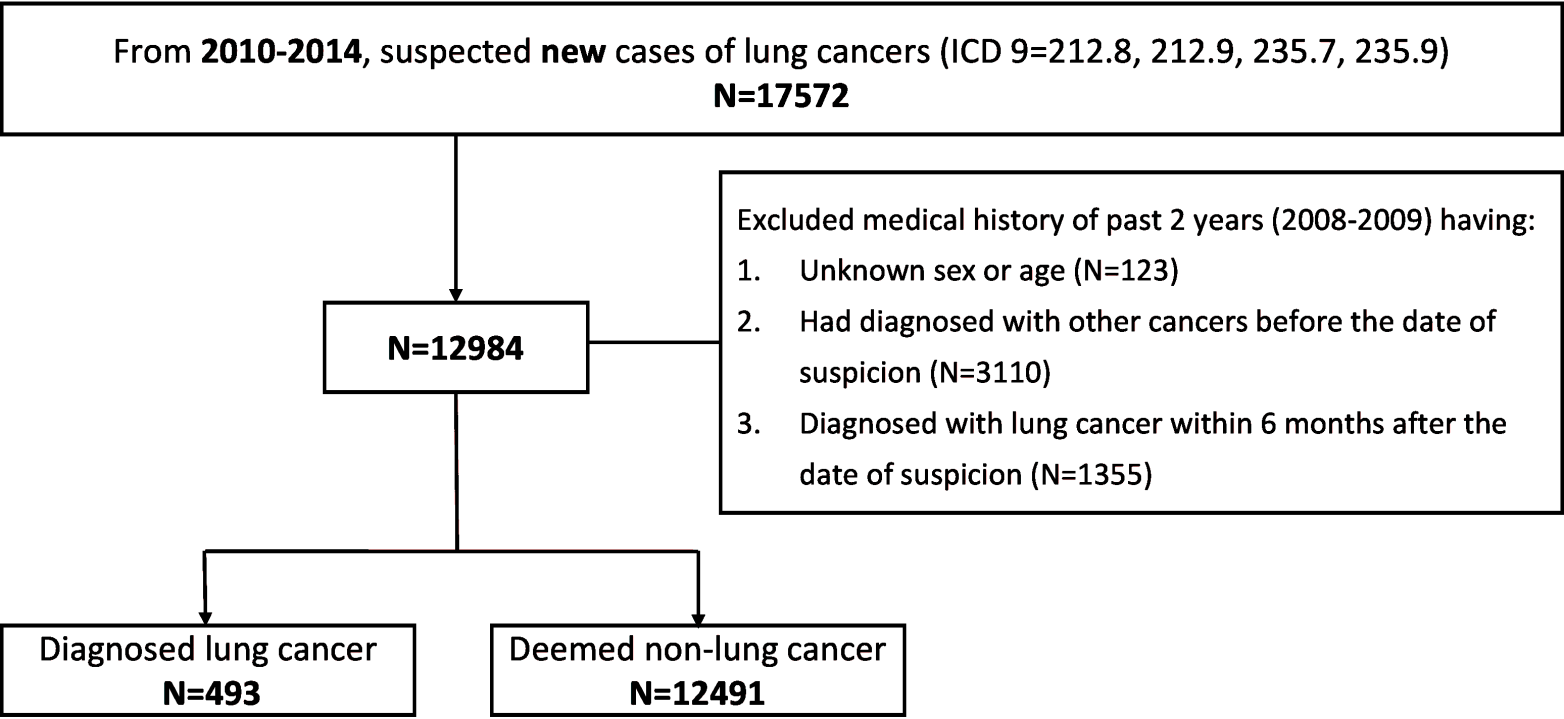
Flowchart demonstrating exclusion criteria and recruited patients. Taiwan’s National Health Insurance Database was used to identify those patients with suspected lung cancer based on ICD code. Those with missing information or a recent cancer diagnosis were excluded. ICD, International Classification of Diseases.

Between January 2010 and December 2014, 17,572 suspected new cases of lung cancer were identified and assigned the appropriate ICD codes. Of those, 4588 were excluded due to insufficient information, confirmed lung cancer within 6 months, and other cancers diagnosed within 2 years. Ultimately, 12,984 participants were included in the study, 493 of which received a confirmed lung cancer diagnosis (**Figure 1**). The ratio of diagnosed lung cancer cases to negative cases was 1:25, or a rate of 3.8%.

Women had a greater incidence of lung cancer than men (7.78; 95% CI, 6.83-8.81 vs 6.25; 95% CI, 5.50-7.08 cases per 1000 person-years). Likewise, those aged more than 65 years had a higher incidence than younger patients (12.68; 95% CI, 11.31-14.17 vs 3.90; 95% CI, 3.35-4.51 cases per 1000 person-years). Of the 493 positive cases, 209 (42.4%) were clinical stage IV, and 153 (31.0%) were stage 0 or I (**Table 1**). In addition, 87/153 cases in stage I were detected with repeated LDCT≥3. Finally, 224 cases (45.4%) were detected after 3 or more LDCT scans, whereas all others were detected after less than 3 scans.

**Table 1:** Distribution of lung cancer cases by clinical stage across sex, age, and number of CT scans.

(per 1000 PY)	No. of cases	stage 0/IA	stage IB	stage IIA/IIB	stage IIIA	stage IIIB	stage IV	unknown	P-value
Overall	493								
Female	244 (49.49%)	70 (28.69%)	21 (8.61%)	5 (2.05%)	12 (4.92%)	8 (3.28%)	105 (43.03%)	23 (9.42%)	0.0042
Male	249 (50.51%)	38 (15.26%)	24 (9.64%)	18 (7.23%)	16 (6.43%)	16 (6.43%)	104 (41.77%)	33 (13.24%)	
Age ≤ 65 y	182 (36.91%)	72 (39.56%)	18 (9.89%)	2 (1.10%)	12 (6.59%)	8 (4.40%)	59 (32.42%)	11 (6.03%)	<0.0001
Age > 65 y	311 (63.09%)	36 (11.58%)	27 (8.68%)	21 (6.75%)	16 (5.14%)	16 (5.14%)	150 (48.23%)	45 (11.47%)	
LDCT < 3	269 (54.56%)	37 (13.75%)	29 (10.78%)	17 (6.32%)	15 (5.58%)	15 (5.58%)	118 (43.87%)	38 (14.12%)	0.0003
LDCT ≥ 3	224 (45.44%)	71 (31.70%)	16 (7.14%)	6 (2.68%)	13 (5.80%)	9 (4.02%)	91 (40.63%)	18 (8.03%)	

LDCT, low-dose computed tomography scan.

**Figure 2:**
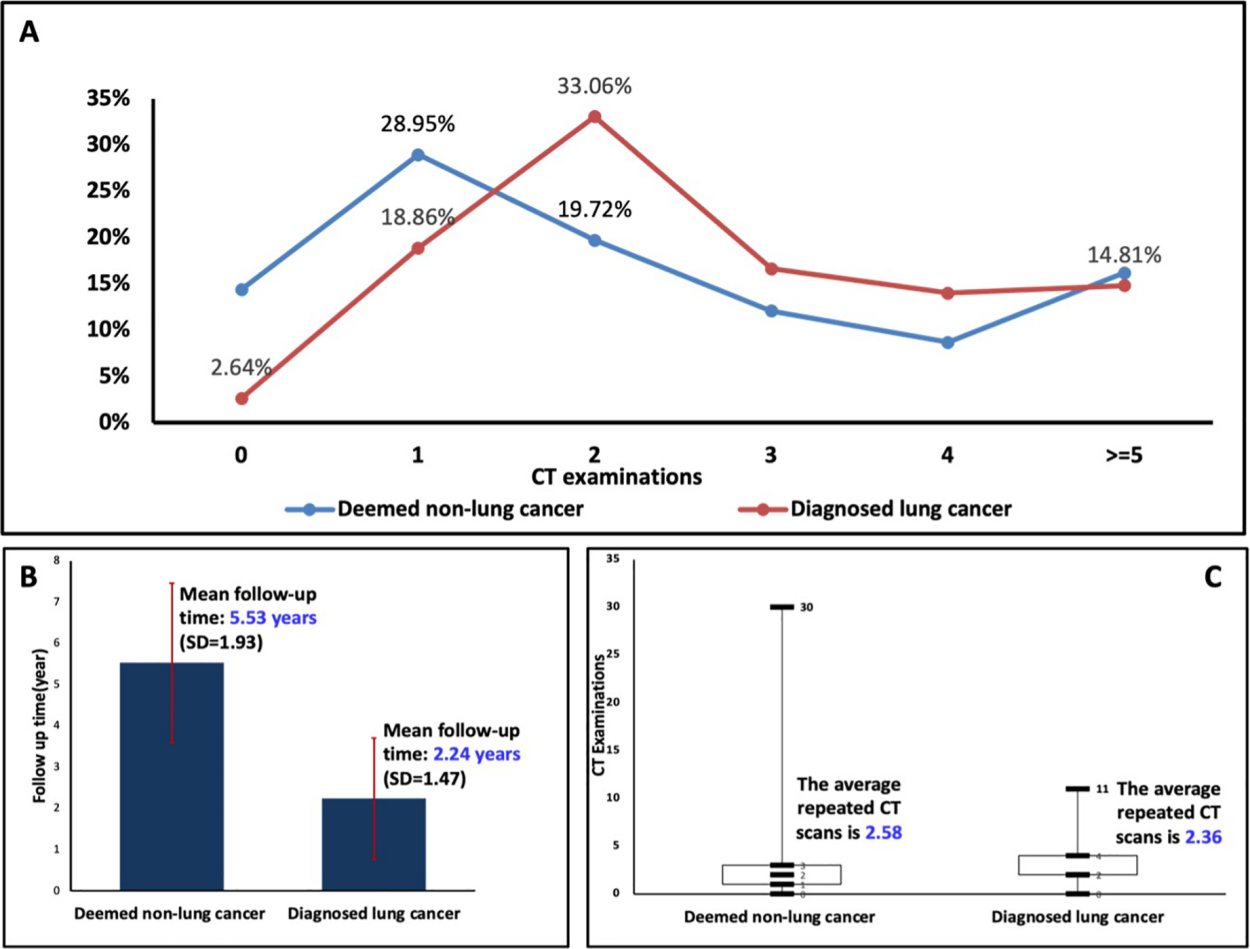
**(A)** Distribution of computed tomography (CT) exams in those diagnosed with lung cancer vs those deemed to be lung cancer free. **(B, C)** Follow-up time and number of CT scans used by those diagnosed with lung cancers vs those deemed to be lung cancer free. SD, standard deviation.

Overall, LDCT scans were repeated about two times during the study period (**Figure 2A**). Of those diagnosed with lung cancer, less than half underwent LDCT more than three times (224 vs 269 or 45.4%). 33.1% of those who were ultimately diagnosed with lung cancer underwent two LDCT scans compared to 19.7% of those who were not diagnosed during this period. The average follow-up time until diagnosis for those ultimately found to have lung cancer was 2.24 (95%CI, 2.11-2.37) years, and required an average of 2.36 (95%CI, 2.20-2.51) CT scans at an average cost of US $397 per scan. Comparatively, those never diagnosed with lung cancer were followed for an average of 5.53 (95%CI, 5.36-5.72) years and underwent an average of 2.58 (95% CI, 2.35-2.81) CT scans at an average cost of $352 per scan (**Figures 2B, C**). The total cost of CT scanning for all patients with suspected lung cancer was $1,429,777, resulting in an average cost of $8913 for each lung cancer diagnosis.

## 4 Discussion

In Taiwan, LDCT screening is not a benefit covered by NHI. It is essential to the efficient allocation of resources and to the potential need for NHI funding for subsequent LDCT examinations when the initial self-paid LDCT screening give positive or non-conclusive results. Our result suggests that more repeated LDCTs may detect more stage I cancers, potentially leading to survival benefits. In addition, we focused our study on real-world patients with suspected lung cancers who were diagnosed with LDCT in subsequent examinations, which inherently leads to higher detection rates.

Smoking rates among women in European and American countries are higher than those in Asia (9). A systematic review based on 13 studies of LDCT screening for lung cancer revealed that among Asian women, the relative risk of a lung cancer diagnosis was similar for smokers and never-smokers (10). The Taiwan Lung Cancer Screening in Never-Smokers Trial (2015 to 2019) showed that female sex, a family history of lung cancer, and age >60 years significantly increased the risk of lung cancer (6). This study did not include screening data for high-risk persons by LDCT. However, our data are crucial for the effective allocation of resources and for NHI funds that may be required for subsequent examinations if initial examinations reveal positive or inconclusive results. With this insight, policy makers can optimize future government-funded LDCT screening programs to ensure that they are effective and accessible to the population. In the present study, the average cost of repeated CT scans for suspected lung cancer was approximately $8913 per person. Our results focus on the costs of repeated LDCTs, without taking into account the survival benefit, and further research is needed to explore this issue. Yuan et al. (11) reported that the optimal age for screening Chinese smokers is 50 years old, at a cost of $12,547 per quality-adjusted life year obtained from the screening. These evidences may support the plan for government-funded LDCT screening programs for high-risk groups, as demonstrated by the Taiwan Lung Cancer Screening in Never-Smokers Trial, and the continued reimbursement of the NHI for the subsequent necessary LDCTs, which costs remain relatively low.

Our study has some limitations. First, the participants were referred due to suspected lung cancers, introducing a self-selection bias that could have influenced the study outcomes. Second, the NHI database has insufficient information about potential confounders such as health literacy, education level, tobacco smoking status, immunotherapy use, cancer subtypes, and mutation status. Furthermore, the study did not provide data on lung cancer mortality and, after suspected lung cancer, subsequent LDCT could not conclude the benefits of survival. Finally, the generalization of our results is limited by the possibility of selection biases among participants who can afford screening.

## 5 Conclusion

Patients with suspected lung cancer were diagnosed after an average follow-up of 2.24 years and underwent an average of 2.36 repeated CT scans. NHI expenditures incurred due to the use of CT scans to follow suspected lung cancer cases remains relatively low.

## Data Availability

The raw data supporting the conclusions of this article will be made available by the authors, without undue reservation.
